# Transcriptional changes induced by candidate malaria vaccines and correlation with protection against malaria in a human challenge model

**DOI:** 10.1016/j.vaccine.2015.07.087

**Published:** 2015-09-29

**Authors:** Susanna Dunachie, Tamara Berthoud, Adrian V.S. Hill, Helen A. Fletcher

**Affiliations:** a The Jenner Institute, Nuffield Department of Medicine, University of Oxford, Churchill Hospital, Oxford OX3 7LJ, UK; b Mahidol-Oxford Tropical Medicine Research Unit, 3rd Floor, 60th Anniversary Chalermprakiat Building, 420/6 Ratchawithi Road, Bangkok 10400, Thailand; c Centre for Tropical Medicine and Global Health, Nuffield Department of Medicine Research Building, University of Oxford, Old Road Campus, Roosevelt Drive, Oxford OX3 7FZ, UK; d London School of Hygiene & Tropical Medicine, London W1CE 7HT, UK

**Keywords:** *Plasmodium falciparum*, Malaria, Vaccine, Microarray, Biomarkers

## Abstract

**Introduction:**

The complexity of immunity to malaria is well known, and clear correlates of protection against malaria have not been established. A better understanding of immune markers induced by candidate malaria vaccines would greatly enhance vaccine development, immunogenicity monitoring and estimation of vaccine efficacy in the field. We have previously reported complete or partial efficacy against experimental sporozoite challenge by several vaccine regimens in healthy malaria-naïve subjects in Oxford. These include a prime-boost regimen with RTS,S/AS02A and modified vaccinia virus Ankara (MVA) expressing the CSP antigen, and a DNA-prime, MVA-boost regimen expressing the ME TRAP antigens. Using samples from these trials we performed transcriptional profiling, allowing a global assessment of responses to vaccination.

**Methods:**

We used Human RefSeq8 Bead Chips from Illumina to examine gene expression using PBMC (peripheral blood mononuclear cells) from 16 human volunteers. To focus on antigen-specific changes, comparisons were made between PBMC stimulated with CSP or TRAP peptide pools and unstimulated PBMC post vaccination. We then correlated gene expression with protection against malaria in a human *Plasmodium falciparum* malaria challenge model.

**Results:**

Differentially expressed genes induced by both vaccine regimens were predominantly in the IFN-γ pathway. Gene set enrichment analysis revealed antigen-specific effects on genes associated with IFN induction and proteasome modules after vaccination. Genes associated with IFN induction and antigen presentation modules were positively enriched in subjects with complete protection from malaria challenge, while genes associated with haemopoietic stem cells, regulatory monocytes and the myeloid lineage modules were negatively enriched in protected subjects.

**Conclusions:**

These results represent novel insights into the immune repertoires involved in malaria vaccination.

## Highlights

• Malaria remains one of the world's major killers.
• Partially effective vaccines against malaria are in development.
• We profiled global gene expression after two candidate vaccine regimens.
• Key pathways of vaccine response include interferon induced genes and the proteasome.
• Global immune profiling approaches are necessary to improve candidate malaria vaccines.

## 1 Introduction

Malaria remains one of the world's greatest killers [1,2], and a vaccine is urgently required. The complexity of immunity to malaria is well known, and clear correlates of protection against malaria have not been established. A better understanding of immune markers induced by candidate malaria vaccines would greatly enhance vaccine development, immunogenicity monitoring and estimation of vaccine efficacy in the field. Systems biology approaches using gene expression profiling to identify early gene signatures associated with vaccine immunogenicity are being pioneered for other pathogens [3–5].

The current leading vaccine against malaria is the RTS,S/AS01B vaccine, a protein-in-adjuvant vaccine which targets the pre-erythrocytic stage circumsporozoite protein. A phase three trial showed an efficacy against first episode of clinical malaria of 46% in children in the first 18 months [6] with efficacy in infants was less at 27%. The predominant immune response induced is antibodies [7,8] but vaccination mediated CD4+ T-cell responses also occur [9–11].

We have previously reported complete or partial efficacy against experimental sporozoite challenge by several vaccine regimens in healthy malaria-naïve subjects in Oxford. These include a prime-boost regimen with RTS,S formulated with an alternative adjuvant AS02A, and modified vaccinia virus Ankara (MVA) expressing the CSP antigen [12], and a DNA-prime, MVA-boost regimen expressing the ME TRAP antigens [13]. Samples from these trials provided the opportunity to perform transcriptional profiling, allowing a global assessment of responses to vaccination.

A greater understanding of the immune response induced by vaccines with partial efficacy, and differences between responders and non-responders is essential to allow further improvements in vaccine design. This study presents results of transcriptional profiling of the malaria vaccine response in a series of early phase malaria vaccine trials where vaccine efficacy was assessed using a human malaria challenge model. We sought to identify key pathways upregulated in response to vaccination with regimens targeting the circumsporozoite protein (CSP) or the thrombospondin-related adhesive protein (TRAP).

## 2 Materials and methods

### 2.1 Donors and clinical trials of candidate malaria vaccines

Samples used in the study came from healthy adult malaria-naïve volunteers in Oxford, UK who participated in vaccine trials as described previously [12,13]. The studies received ethical approval from the Oxfordshire Research Ethics Committee, Human Subjects’ Protection Committee at PATH (Program for Appropriate Technology in Health) in Seattle, WA, USA, the Naval Medical Research Center Institutional Review Board and the U.S. Navy Surgeon General in accordance with U.S. Navy regulations (SECNAVINST 3900.39B) and in compliance with all applicable U.S. Federal regulations governing the use of human subjects. All participants gave written, informed consent prior to participation. The trial was conducted according to Good Clinical Practice guidelines, was externally monitored, and was approved by the UK Medicines and Healthcare products Regulatory Agency (MHRA).

Subjects in the CSP study [12] received two intramuscular doses of the RTS,S/AS02A vaccine (*GlaxoSmithKline Biologicals, Rixensart, Belgium*) and one intradermal dose of 1 × 10^8^ plaque forming units (pfu) MVA-CS (*Oxxon Therapeutics, Oxford, UK*). RTS,S consists of the carboxyl terminal (a.a. 207–395) of the 3D7 circumsporozoite protein fused to the hepatitis B surface antigen, co-expressed in yeast with the non-fused hepatitis B surface antigen. The proprietary adjuvant AS02A is an oil-in-water emulsion containing the two immunostimulants QS21 and MPL. The vaccine was supplied as a lyophilised pellet with separate adjuvant, and reconstitution gave 50 μg of RTS,S with AS02A in one 0.5 ml dose. MVA-CS is a recombinant virus using the viral vector modified vaccinia virus Ankara. The insert sequence of MVA-CS encodes the entire 3D7 circumsporozoite protein, recoded to mammalian codon bias to facilitate antigen expression. Four subjects studied received two doses of RTS,S/AS02A one month apart followed one month later by MVA-CS (“RRM”), while four subjects received one dose of MVA-CS followed at monthly intervals by two doses of RTS,S/AS02A (“MRR”). As no significant difference was observed in the original study for antibody responses, cellular responses or vaccine efficacy [12] these subjects are analysed as one group.

Subjects in the TRAP study [13] received two intramuscular vaccinations of 2 mg of DNA-ME TRAP (*Oxxon Therapeutics, Oxford, UK*) one month apart followed one month later by one intradermal vaccination of 1.5 × 10^8^ pfu MVA-ME TRAP (*Oxxon Therapeutics, Oxford, UK*) one month later. ME TRAP is a multiple epitope string including 14 CD8 T-cell epitopes, 1 CD4 T-cell epitope, and 2 B-cell epitopes from six pre-erythrocytic *Plasmodium falciparum* antigens fused to the N terminus of TRAP as previously described [14].

PBMC from eleven subjects in a third study [15] were used to validate the gene expression changes by Q-PCR. Subjects in this trial had received vaccination with FP9-ME TRAP (fowlpox virus 9, *Oxxon Therapeutics, Oxford, UK*)) and MVA-ME TRAP.

The efficacy of the vaccine schedules was assessed by experimental sporozoite challenge, whereby the volunteers were exposed to the bites of five laboratory-reared mosquitoes infected with the chloroquine-sensitive 3D7 strain of *P. falciparum*. For the CSP study, vaccinated subjects underwent sporozoite challenge alongside five unvaccinated control subjects, 28 days after the final immunisation. In this study four out of twelve vaccinated subjects demonstrated complete (sterile) protection against malaria (no parasitaemia detectable within 21 days of challenge) and as a group there was a delay to parasitaemia compared to controls. For the TRAP study eight vaccinated subjects underwent sporozoite challenge alongside six unvaccinated control subjects, 14 days after the final immunisation. In this study one out of eight vaccinated subjects demonstrated complete (sterile) protection against malaria and as a group there was a delay to parasitaemia compared to controls. Across both studies all unvaccinated control subjects developed slide-confirmed malaria at a mean of 11.1 days (range 9–14 days).

### 2.2 Study design for gene expression profiling

The sixteen subjects studied by gene expression profiling in this study are summarised in Table 1a and b. Eight subjects were chosen from the CSP study and eight from the TRAP study. 14/16 underwent experimental sporozite challenge and all PBMC in this study were drawn on the day of experimental sporozoite challenge prior to challenge (28 days after final vaccination for CSP study and 14 days after final vaccination for TRAP study). Each study was analysed separately and then comparisons between the two studies were made. The two studies were then combined, with gene expression signal for vaccine antigen-stimulated cells for each sample normalised to its unstimulated pair.

### 2.3 Preparation of PBMC and cell stimulation

Peripheral blood mononuclear cells (PBMC) were isolated by density gradient centrifugation, cryopreserved then thawed when required as previously described [16]. PBMC were stimulated with CSP (for the eight subjects in the CSP study) or TRAP (for the eight subjects in the TRAP study) peptide pools for 12 h overnight at a concentration of 2 μg/ml. The CSP pool consisted of 61 15-mer peptides and the TRAP pool consisted of 57 20-mer peptides. PBMC incubated with media alone were control (unstimulated) cells. After 12 h the cells were spun and resuspended in RLT buffer (*Qiagen, Crawley, West Sussex, UK*) containing beta-mercaptoethanol (*VWR, Lutterworth, Leicestershire, UK).*

### 2.4 Preparation of RNA for arrays

RNA extraction was performed using the RNeasy Mini-kit kit. (*Qiagen, Crawley, West Sussex, UK*) according to the manufacturer's instructions, including an on-column DNAse treatment. A median of 0.37 μg (range 0.19–0.52 μg) RNA was obtained from 1 × 10^6^ PBMC. RNA samples for arrays were amplified using the Illumina TotalPrep RNA Amplification kit (*Ambion, Austin, TX*) which is based on the Eberwine protocol [17] and incorporates a Biotin-16-UTP label into the amplified RNA. RNA yield was quantified using a NanoDrop ND 1000 Spectrophotometer (*NanoDrop Technologies, Wilmington, DE*) and the quality of the samples was checked as satisfactory using an Agilent Bioanalyzer 2100 (*Agilent Technologies, Santa Clara, CA*). Amplification according to the manufacturer's instructions gave a median yield of 12.7 μg (range 2.0–35.9 μg).

### 2.5 Microarray procedures

Amplified RNA (1 μg per array) was hybridized to the Illumina HumanRefSeq-8 BeadChip according to the manufacturer's instructions (*Illumina, San Diego, CA, USA*). The HumanRefSeq-8 bead chip comprises of 24,000 sequences representing 16,238 genes from the curated portion of the NIH Reference Sequence Database http://www.ncbi.nlm.nih.gov/RefSeq/. Each sequence is represented at least 30 times on the array. Arrays were scanned with an Illumina bead array reader confocal scanner, according to the manufacturer's instructions. Array data processing and preliminary analysis with background subtraction was performed using Illumina BeadStudio software.

### 2.6 Gene expression data analysis

Gene expression analysis was performed using Genespring GX version 7.3.1 (*Agilent Technologies, Santa Clara, CA*). Data were normalised in Genespring GX by per chip to 50th percentile, per gene to the median, and values less than 0.01 were reset to 0.01. For some of the analysis samples were normalised per sample by normalising each stimulated sample to its unstimulated pair. Differential expression was assessed on the Genespring normalised data by Welch *T*-test, a parametric test not assuming equal variances, in Genespring GX. Lists of differentially expressed genes between conditions were analysed according to Gene Ontology (GO) categories (Genespring) and using *Pathway Express* [18]. In the *Pathway Express* program an enrichment analysis based on a hypergeometric distribution identifies pathways containing a proportion of differentially expressed genes that is significantly different from what is expected by chance. A perturbation factor PF(g) is also calculated for each gene on each pathway using the foldchange in gene expression and the number and its position on the pathway. Correction for multiple testing was using the False Discovery Rate method.

For analysis of protection against malaria, data on days to parasitaemia was used for all fourteen subjects who underwent sporozoite challenge. A relative delay in time to parasitaemia compared to unvaccinated control subjects or unprotected subjects represents partial protection, as calculated by using highly sensitive qPCR to estimate vaccine-induced reduction in the number of parasites emerging from the liver [19]. For the purpose of analysis fully protected subjects were designated as 21 days to parasitaemia as follow-up ended at 21 days. Spearman's rank correlation test in Genespring was used to identify genes whose expression correlated with protection against malaria by using days to parasitaemia as a continuous parameter, with a two-tailed *P* < 0.01. In addition, for gene set enrichment analysis (below), a comparison in gene expression was made for the three subjects completely protected against malaria versus the eleven subjects who developed malaria. For this analysis, each peptide stimulated PBMC sample was normalised to its unstimulated pair prior to comparison.

Further statistical analysis was performed by Gene Set Enrichment Analysis (GSEA), using the Molecular Signatures Database, MSigDB http://www.broad.mit.edu/gsea/msigdb/index.jsp [20]. This database is a collection of 1891 gene sets assembled from a range of sources including online pathway databases, PubMed literature and expert opinion. Briefly, genes were ranked according to their differential expression across two conditions and the gene lists generated underwent GSEA analysis to quantify the degree to which the database genesets occur towards the top (up-regulated genes) or towards the bottom (down-regulated genes) of the ranked list of genes from the experiment [21]. GSEA uses a variation of the Kolmogorov–Smirnov test (a statistical test of goodness of fit, and is better than the X2 test for small sample sizes) to give an enrichment score for each geneset. The enrichment scores are normalised for the size of the gene sets and correction for multiple testing was by computing the Benjamini and Hochberg false discovery rate [22]. Pathways up or down regulated were displayed by modular mapping, whereby each gene set was assigned to a module using online pathway databases and PubMed literature [23].

Raw data transcripts for all samples are available in Supplement S1.

### 2.7 Real-time quantitative PCR (Q-PCR) analysis

Q-PCR was used to validate the array findings. Reverse transcription of RNA into cDNA was performed using the Omniscript kit. (*Qiagen, Crawley, West Sussex, UK*) according to the manufacturer's instructions. Quantitative real time Reverse Transcription PCR was performed using the Lightcycler 2.0 (*Roche, Basel, Switzerland*) carousel-based system using Quantitect SYBR Green Mastermix (*Qiagen, Crawley, West Sussex, UK*). All reactions were performed in duplicate with two negative controls per run. Data were produced as amplification plots with fluorescence plotted against number of cycles. The *C*_*T*_ (threshold cycle) value for each sample was calculated with the threshold set during the log-linear phase of amplification using the “Fit points” method. A selection of up-regulated and down-regulated genes was measured to confirm the array analysis, normalised to the housekeeping gene *HPRT*. Differences between paired samples compared by Wilcoxon's signed rank test using *Graphpad Prism version 5.0*.

## 3 Results

### 3.1 Antigen-specific changes in expression of genes from donors vaccinated with RTS,S/AS02A and MVA-CS (CSP study)

Transcriptional profiles were compared in PBMC stimulated with CSP and paired unstimulated PBMC in samples from eight volunteers 28 days post-vaccination with RTS,S/AS02A and MVA-CS. 128 genes were differentially expressed with the significance level at *P* < 0.01 and 744 genes were differentially expressed with the significance level at *P* < 0.05. There was a predominance of genes thought to be produced chiefly by monocytes in response to IFN-γ including *WARS*, *IRF1* and *STAT1*. The FY gene, known as the Duffy antigen/receptor for chemokine (*DARC*) showed antigen-specific down-regulation. A selection of differentially expressed genes is shown in Table 2.

**Fig. 1:**
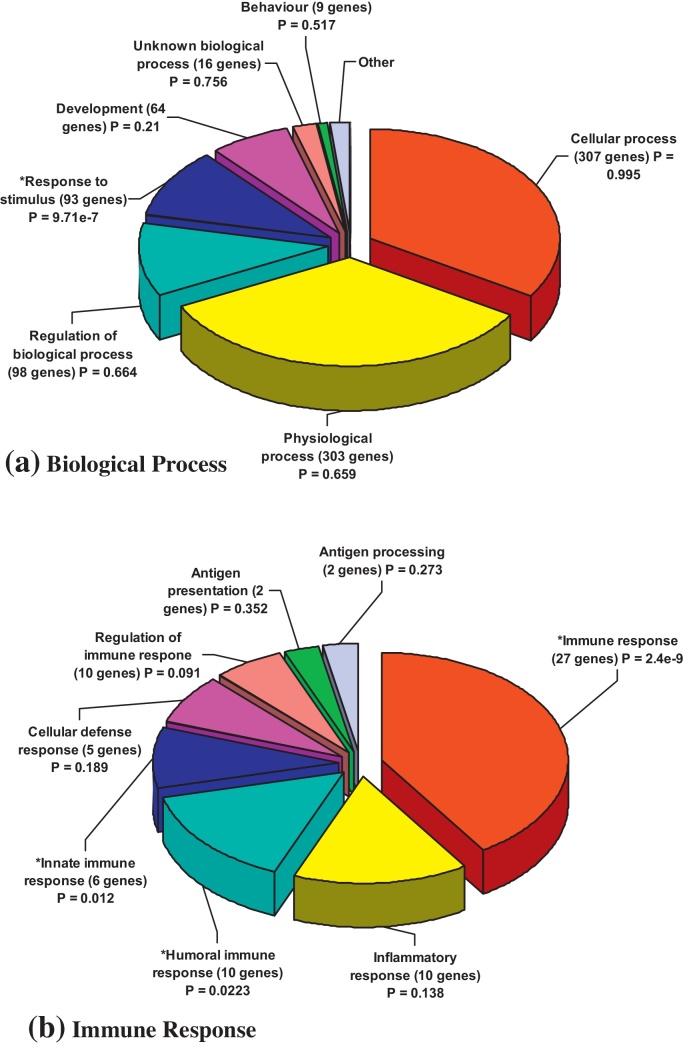
*Gene Ontology processes involved in differentially expressed genes in CSP Study.* The list of 744 genes identified as differentially expressed (Welch *t*-test, *P* < 0.05) between the 8 CSP-stimulated PBMC samples and the 8 paired unstimulated samples post vaccination by Welch *t*-test was used to generate pie charts of gene ontology processes by biological process (a, all 744 genes) and immune response (b, 56 out of 744 genes) in *Genespring*.

The differentially expressed genes in the CSP study were also analysed by gene ontology process (Fig. 1) and by pathways involved, where the pathways are ranked according to both the number of genes present for each pathway compared to the expected number for the total number of genes, and by the expression foldchanges of the genes. The top three pathways involved were the antigen processing and presentation pathway (including up-regulation of *CD74, HLA-DPA1, TAP 1, TAP 2*, and *CIITA*), the Jak-STAT signalling pathway (including up-regulation of *IFN-γ, IL-15, IL-21R, IL-22, IL-26, IL-4R, JAK2, SOC1, SOC2* and *STAT1* and the Phosphatidylinositol signalling system (including down-regulation of *PIK3R1, PIK4CA* and *PIP5K1B*).

### 3.2 Antigen-specific changes in expression of genes from donors vaccinated with DNA-ME TRAP and MVA-ME TRAP (TRAP study)

Transcriptional profiles were compared in PBMC stimulated with TRAP and paired unstimulated PBMC in samples from eight volunteers 14 days post-vaccination with DNA-ME TRAP and MVA-ME TRAP. 86 genes were differentially expressed with the significance level at *P* < 0.01 and 526 genes were differentially expressed with the significance level at *P* < 0.05. A selection of the genes with *P* < 0.01 is shown in Table 3. There was a remarkable degree of overlap between the genes up-regulated by CSP stimulation for CSP study PBMC and the genes up-regulated by TRAP stimulation for TRAP study PBMC, such that 43 out of the 56 genes (77%) up-regulated at least 1.5 fold in the CSP samples were also up-regulated at least 1.5 fold in the TRAP samples.

**Fig. 2:**
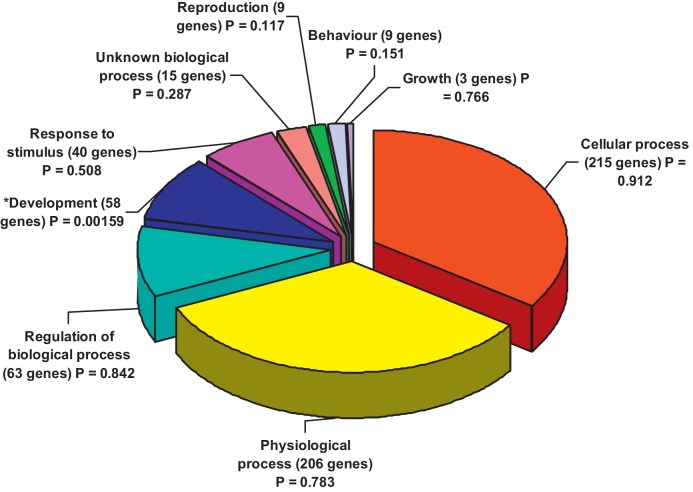
*Gene Ontology processes involved in PBMC differentially expressed genes, TRAP-stimulated PBMC compared to unstimulated PBMCs post-vaccination.* The list of 526 genes identified as differentially expressed (Welch *t*-test, *P* < 0.05) between the 8 TRAP-stimulated PBMC samples and the 8 unstimulated samples post vaccination by Welch *t*-test was used to generate a pie chart of gene ontology processes by biological process (all 526 genes) in *Genespring*. No GO processes for immune response were significant.

The differentially expressed genes were also analysed by gene ontology processes in *Genespring* (Fig. 2) and by pathways involved in *Pathways Express*, where the pathways are ranked as before according to both the number of genes present for each pathway compared to expected for the total number of genes, and by the expression foldchanges of the genes. The top pathway identified was the Antigen Processing and Presentation pathway (one gene *HLA-DQA2*). The second ranked pathway was the Phosphatidylinositol Signaling System (*CALML3, PIP5K3, PLCE1* and *SYNJ1*) and the third was the Colorectal Cancer-related pathway (including *ACVR1C, AKT2, FZD6, MSH2*, and *TGFBR1*)

### 3.3 Relationship between gene expression changes and protection against experimental sporozoite challenge

Fourteen subjects underwent sporozoite challenge, with three fully protected against malaria (two in the CSP study and one in the TRAP study). A Spearman's rank test was performed using all the peptide stimulated PBMC samples (each normalised to its unstimulated pair) from both the CSP and the TRAP study post-vaccination treated as one experiment, and gene expression correlated with the number of days to parasitaemia. 292 genes were identified (*P* < 0.01, no genes identified after adjusting for multiple corrections). This gene list was analysed by *Pathways Express* and the top ranking pathway was Leukocyte transendothelial migration (including *CLDN15, CYBB, PIK3R5, PRKCA, SIPA1* and *VAV1*) with the Calcium Signalling Pathway and the Natural Killer Cell Mediated Cytotoxicity pathway ranked second and third, respectively, as shown in Table 4.

Because of the small numbers involved, it was difficult to look for the relationship between differential gene expression and protection against malaria for the CSP and TRAP studies separately. However it was noted that for the two subjects in the CSP study with sterile protection against malaria, 703 genes were differentially expressed at the significance level at *P* < 0.05 compared to the four subjects who developed malaria (comparing CSP-stimulated PBMC normalised to each unstimulated pair), including upregulation of *IL17F, IGJ* and *IL13*.

### 3.4 Modular approach to gene set enrichment analysis

**Fig. 3:**
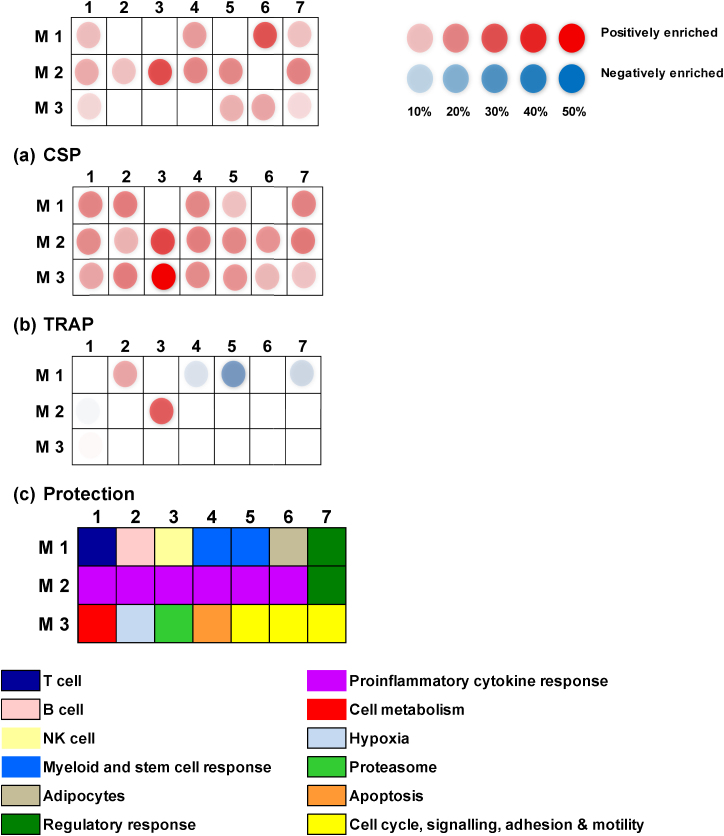
*Modular representation of differentially expressed genes.* (a) CSP. A number of modules were enriched in CSP-stimulated PBMC compared to unstimulated PBMC from subjects in the CSP study, including *Interferon Induced* and *Adipocytes.* No modules were negatively enriched. (b) TRAP. A number of modules were enriched in TRAP-stimulated PBMC compared to unstimulated PBMC from subjects in the TRAP study, including *Proteasome* and *Interferon Induced.* No modules were negatively enriched. (c) Protection. When the three subjects (two from CSP study, one from TRAP study) who did not develop malaria were compared to the eleven subjects who did develop malaria, a number of modules were both positively enriched (Interferon Induced and Antigen Presentation) and negatively enriched (*Haemopoetic stem cell, Regulatory monocytes* and *Myeloid*).

To further explore the biological meaning of the differentially expressed genes in this dataset, GSEA was performed (FDR 1%) and genesets were classified into modules (Table 5 and Fig. 3). When PBMC stimulated with peptides were compared to paired unstimulated PBMC, a number of pathways were positively enriched, but significant downregulation of pathways was not seen. For the CSP study, the module with the greatest enrichment was *Interferon induced* (30% of all genes in the *Interferon induced* module were upregulated) followed by *Adipocytes* module (29% of genes were upregulated). For the TRAP study there was a marked enhancement of the *Proteasome* module (72% of genes). The second module with enhancement was *Interferon induced* (32% of genes).

In order to facilitate identification of pathways involved in protection against malaria, a comparison in gene expression was made for the three subjects completely protected against malaria versus the eleven subjects who developed malaria. For this analysis, each peptide stimulated PBMC sample was normalised to its unstimulated pair prior to comparison. The top two modules upregulated in protected subjects compared to unprotected subjects were *Interferon induced* (27% of genes) and *Antigen Presentation* (14% of genes). Three related modules were downregulated in protected subjects: the *HSC* module (Haemopoetic stem cell, 32% of genes), *Regulatory Monocytes* module (16% of genes) and *Myeloid Lineage* module (15% of genes).

### 3.5 Confirmation of expression changes by Q-PCR

**Fig. 4:**
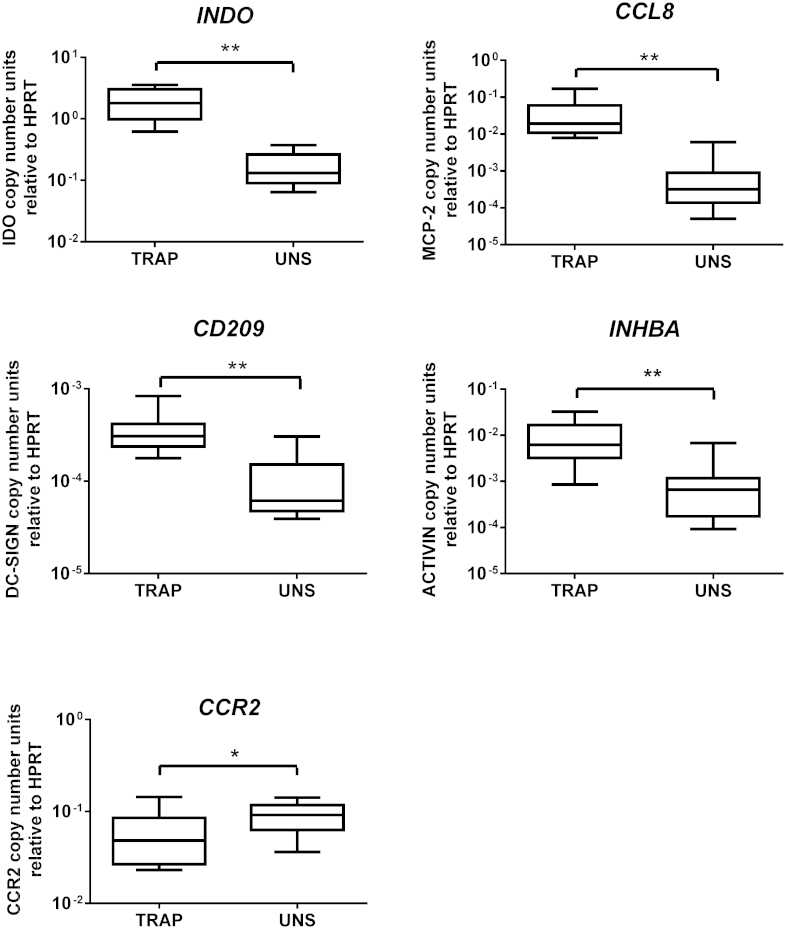
*Confirmation of gene expression changes by Q-PCR.* The expression levels of 5 genes were compared in TRAP stimulated and unstimulated PBMC in an independent set of samples from subjects in a different vaccine trial receiving a TRAP-containing regimen.

The changes in expression of a selection of genes identified in the array experiments were confirmed by Q-PCR in an independent dataset of eleven PBMC samples drawn from subjects who had also received vaccine regimens encoding TRAP. For all 5 genes checked, the same relationship of upregulation by TRAP stimulation (for *INDO*, *CCL8, CD209* and *INHBA*) or downregulation (*CCR2*) was confirmed (Fig. 4).

## 4 Discussion

The aim of the gene expression studies was to exploit a unique resource of samples from malaria vaccine trials to study antigen-specific responses before and after vaccination. The availability of sporozoite challenge data allowed the opportunity to examine for relationships between expression changes and protection against malaria.

Many of the genes identified as upregulated and/or differentially expressed such as *CCL8, WARS* (tryptophanyl-tRNA synthetase), *INDO* (indoleamine 2,3-dioxygenase) and *CXCL10* are known to be in the IFN-γ pathway and are thought to arise predominantly from monocytes. Both pathway analysis and GSEA with module allocation identified IFN induced genes as a theme for response to CSP stimulation for CSP vaccinated subjects, for response to TRAP stimulation for TRAP vaccinated subjects, and a role in protection against malaria. This may reflect a cascade of events set in motion by antigen recognition by T-cells, representing the activation of monocytes by antigen-specific IFN-γ. IFN-γ is well established as a surrogate marker of T-cell immunogenicity by our laboratory and others for both natural immunity against malaria [24–26] and response to vaccination against malaria [13,27–29]. Interestingly IFN-γ itself did not always feature as differentially expressed on the arrays, despite detection of up-regulation by Q-PCR. This suggests that arrays have a lower dynamic range than Q-PCR for the detection of fold change differences in expression. In agreement with the results presented here, a study of gene expression changes in both early malaria, using PBMC samples from volunteers in a sporozoite challenge study, and in established malaria in adults in Cameroon [30] showed induction of IFN-γ pathways including *STAT1* and *JAK2* kinases in both groups compared to malaria naïve subjects. Another study looked at gene expression in Kenyan children with acute malaria [31], but studied whole blood responses and most identified changes were linked to erythrocytes and neutrophils. However up-regulation of IFN-γ related genes such as *HM74* and *WARS* was reported.

It was difficult to detect significant changes in gene expression at the individual gene level. This is likely due to the small sample size as only a low number of volunteers were vaccinated and challenged in early phase clinical trials, where each regimen is unique. In addition, the impact of vaccination on global gene expression is likely to be more subtle than malaria disease where an individual can have chronic antigen exposure and a high parasite burden. However the expression changes were confirmed for all five genes examined in samples from a different vaccine regimen trial. There was a substantial overlap in results for both the CSP study and the TRAP study, for example 77% of the genes up-regulated in the antigen-stimulated PBMCs compared to unstimulated PBMCs post-vaccination in the CSP study were up-regulated in the TRAP study. This is in spite of different antigens and vaccine types, and lends cross-validity to the findings.

The results give an insight into immune responses at a general level across PBMCs. Many of the genes whose expression changes following antigen stimulation are thought to arise from monocytes, yet flow cytometry analysis demonstrated that CD14+ cells comprised less than 6% of all cells (unpublished findings), thus exerting a very dominant effect and being the responders for antigen recognition upstream.

An antigen-specific up-regulation in IL-13 was observed post-vaccination in the two CSP study subjects who were completely protected against malaria following vaccination with the antibody-inducing vaccine RTS,S/AS02A along with MVA-CS. An association between an IL-13 polymorphism linked with higher IL-13 levels and protection against severe malaria has been reported [32], and case-control studies in Gabon have suggested a role for IL-13 in the control of malaria infection, speculatively by acting alongside IL-4 to provide B cell help in switching to specific IgG1 antibody production [33]. IL-13 was up-regulated in the TRAP subjects, although the TRAP regimen is primarily an inducer of cellular immunity. Expression of IL13AR2 was associated with longer days to parasitaemia.

At the pathways level changes in antigen presentation and processing, the Jak–Stat pathway and the phosphatidylinositol signalling system emerged as the key pathways invoked by antigen stimulation after vaccination. The presence of the colorectal cancer pathway is explained by the non-specific inflammatory genes involved such as *ACVR1C, AKT2, FZD6, MSH2*, and *TGFBR1.* For the CSP study a number of upregulated genes corresponded to the Adipocytes module, which features many immune related genes including *CD53*, *HCLS1* and *IFI30.*

It is of interest that subjects vaccinated with TRAP containing regimens showed a marked enrichment in activity of genes related to the proteasome. A gene expression study of 39 subjects receiving RTS,S vaccine reported upregulation of genes in the proteasome degradation pathway (*PSME2, PSMB9, PSMB6*,and *PSMA4*) to be associated with protection [34]. Similar strong induction of the proteasome module has been reported in a whole-blood transcriptome study of *P. falciparum*-infected West African children alongside a *Plasmodium chabaudi* mouse model [35]. The proteasome plays a central role in MHC peptide processing and antigen presentation [36] and vaccine strategies that promote activation of the proteasome should be pursued.

Sterile protection against malaria was associated with positive enrichment of genes associated with IFN induction and antigen presentation modules, and negative enrichment of genes associated with haemopoietic stem cells, regulatory monocytes and the myeloid lineage modules. The downregulation of stem cell precursors and myeloid lineage is compatible with recent literature showing a correlation between low ratio of monocyte to lymphocyte count in the differential blood count of African children and both improved response to RTS,S vaccine [37] and decreased susceptibility to malaria [38].

For the analysis of protection against malaria challenge, we acknowledge the limitations of combining results from the two different vaccine studies. However because of the small numbers involved in these studies and the limited existing published data we combined the two studies to seek to identify the common themes associated with protection from infection by candidate malaria vaccines. It is reasonable to assume that the complete sterile protection in all three cases was due to vaccine-induced changes, because historically all unvaccinated control subjects have become infected upon sporozoite challenge. Our experimental design using antigen stimulation allowed us to focus on vaccine-specific changes. We analysed each of the two regimens separately, and then looked for common themes between the two regimens. There was a surprisingly high degree of overlap in differentially expressed genes in response to stimulation with the vaccine antigens between the two studies and we therefore believe combing the two studies is justified.

The results of these array experiments confirm and extend existing published transcriptomics studies of response to infection and vaccination. A study of transcriptional changes in PBMCs following vaccination with smallpox, vaccination with yellow fever or natural upper respiratory tract infection [39] reported up-regulation of many IFN-stimulated genes with a particular predominance of genes involved in proteolysis and antigen presentation such as *CD74* and *LAP3*. Another study of transcriptome changes in PBMC in response to yellow fever vaccination confirmed upregulation of IFN-induced and anti-viral genes [4]. A small study examining antigen-specific responses to influenza vaccination also reported activation of pathways relating to IFN-γ and antigen presentation [40]. A larger study of transcriptome changes in PBMC following influenza vaccination compared to baseline showed induction of IFN-related genes by a live attenuated influenza vaccine but not by an inactivated vaccine [3].

## 5 Conclusions

The samples from two malaria vaccine trials have offered the opportunity to profile antigen-specific responses at the transcript level. The findings confirm and extend knowledge on responses to vaccination and infection at the molecular level, and are informative in elucidating which pathways vaccination strategies target. Vaccine strategies that enhance activation of the proteasome and promote a switch towards lymphoid lineage are likely to be important. Further studies in larger datasets will consolidate these findings.

## Conflict of interest statement

None declared.

## Figures and Tables

**Table 1:** Characteristics of the PBMC samples for gene expression.

(a)				
Vaccine group	Total	Challenged	Sterile protection by challenge	No sterile protection by challenge
CSP	8	6	2	6
TRAP	8	8	1	7
Total	16	14	3	11

(b)					
Subject	Vaccine regimen	Anti-CS antibody response (μg/ml)	Ex vivo ELISPOT 3rd Vac + 28 (SFC/10^6^ PBMC)	Days post vaccination	Protection against challenge
CSP-1	RRM	22	6	14	Fully protected
CSP-2	RRM	30	51	14	Parasitaemia day 13.5
CSP-3	RRM	23	38	14	Parasitaemia day 12.5
CSP-4	RRM	50	8	14	Fully protected
CSP-5	MRR	98	75	14	Not challenged
CSP-6	MRR	5	28	14	Parasitaemia day 12.5
CSP-7	MRR	72	20	14	Not challenged
CSP-8	MRR	70	71	14	Parasitaemia day 12.5
Subject	Vaccine regimen	Anti-TRAP antibody (geometric mean titre relative to pre-vaccination)	Ex vivo ELISPOT 3rd Vac + 14 (SFC/10^6^ PBMC)		Protection against challenge
TRAP-1	DDM-ME TRAP	83	14	28	Parasitaemia day 9
TRAP-2	DDM-ME TRAP	38	205	28	Parasitaemia day 12.5
TRAP-3	DDM-ME TRAP	51	468	28	Parasitaemia day 12.5
TRAP-4	DDM-ME TRAP	35	64	28	Parasitaemia day 14
TRAP-5	DDM-ME TRAP	409	341	28	Parasitaemia day 11
TRAP-6	DDM-ME TRAP	102	106	28	Parasitaemia day 12
TRAP-7	DDM-ME TRAP	92	749	28	Parasitaemia day 11.5
TRAP-8	DDM-ME TRAP	120	421	28	Fully protected

Part a shows the number of subjects whose PBMC samples were studied for gene expression profiling, and the number of subjects who underwent sporozoite challenge. Part b shows the details of vaccine regimen, cellular immune response, antbody response, time of sample and results of sporozoite challenge for each subject. CSP = circumsporozoite protein, TRAP = thrombospondin related adhesive protein, SFC/10^6^ PBMC = spot forming cells per million peripheral blood mononuclear cells, *R* = RTS,S/AS02A vaccine, *M* = MVA-CS vaccine, RRM = two dose of RTS,S/AS02A vaccine followed by one dose of MVA-CS vaccine, MRR = one dose of MVA-CS vaccine followed by two doses of RTS,S/AS02A vaccine. DDM-ME TRAP = two doses of DNA-ME TRAP followed by one dose of MVA-ME TRAP.

**Table 2:** Genes differentially expressed in CSP-stimulated PBMC compared to unstimulated PBMC.

Gene name	Description	*P* value	Median foldchange	Minimum foldchange	Maximum foldchange
TNFAIP2	Tumor necrosis factor, alpha-induced protein 2	0.00165	1.9	1.2	3.6
FY	Duffy blood group	0.00193	0.9	0.8	1.0
SOCS1	Suppressor of cytokine signaling 1	0.0022	1.5	1.1	2.9
IRF1	Interferon regulatory factor 1	0.00238	1.8	1.0	3.7
WARS	Tryptophanyl-tRNA synthetase	0.00278	2.3	1.1	7.8
P2RY6	Pyrimidinergic receptor P2Y, G-protein coupled 6	0.0028	1.5	1.1	3.5
IL4R	Interleukin 4 receptor	0.00285	1.4	1.0	1.8
CASP7	Caspase 7 apoptosis-related cysteine protease	0.00325	1.3	1.1	1.6
GBP5	Guanylate binding protein 5	0.00355	2.5	1.2	11.4
LAP3	Leucine aminopeptidase 3	0.00674	2.0	1.3	6.9
GBP4	Guanylate binding protein 4	0.0087	1.7	1.2	5.0
STAT1	Signal transducer and activator of transcription 1	0.00923	2.2	1.1	9.5

This is a selection of genes differentially expressed in the group of 8 CSP stimulated PBMC samples from the CSP study compared to the 8 unstimulated paired samples, from the post vaccination timepoint, analysed by Welch *t*-test, *P* < 0.01.

**Table 3:** Genes differentially expressed in TRAP-stimulated PBMC compared to unstimulated PBMC.

Gene name	Description	*P* value	Median foldchange	Minimum foldchange	Maximum foldchange
XKRY	X Kell blood group precursor-related, Y-linked	0.00159	1.16	1.04	1.33
HOM-TES-85	HOM-TES-85 tumor antigen; Leucine zipper protein 4	0.0016	1.26	0.97	1.40
MCP-3	Monocyte chemotactic protein-3; CCL7 Chemokine CC motif ligand 7	0.0018	4.24	0.36	15.74
PROC	Protein C	0.00276	0.91	0.82	1.14
TCP11	T-complex homolog	0.00358	0.91	0.79	1.00
PLCL1	Phospholipase C-like 1	0.00427	0.93	0.81	1.14
SGK2	Serum/glucocorticoid regulated kinase 2	0.00428	0.91	0.84	1.08
TSLP	Thymic stromal lymphopoietin (stim DC maturation and Treg)	0.00462	0.91	0.83	1.14
CEACAM3	Carcinoembryonic antigen-related cell adhesion molecule 3	0.005	1.24	1.12	1.38
GPR91	G protein-coupled receptor 91	0.00524	1.24	0.89	1.53
TGFBR1	Transforming growth factor, beta receptor I (activin A receptor type II-like kinase)	0.00587	0.95	0.84	1.08
RNF29	Ring finger protein 29	0.00587	0.98	0.86	1.07
MRGX1	G protein-coupled receptor MRGX1	0.00588	0.97	0.90	1.07
CD164	CD164 antigen, sialomucin	0.00662	1.00	0.93	1.05
LIMK2	LIM domain kinase 2	0.00675	2.84	0.84	4.39
ZFH4	Zinc finger homeodomain 4	0.00724	1.21	1.04	1.37
ICOSL	Inducible T-cell co-stimulator ligand	0.00911	0.96	0.89	1.04
TGM2	Transglutaminase 2 (C polypeptide, protein-glutamine-gamma-glutamyltransferase)	0.00913	1.38	0.99	2.13
FOXP2	Forkhead box P2	0.00941	1.18	1.04	1.34

This is a selection of genes differentially expressed in the group of 8 TRAP-stimulated PBMC samples from TRAP study compared to the 8 unstimulated samples at the post-vaccination timepoint 14 days after the final vaccine, day of challenge (DOC), analysed by Welch *t*-test, *P* < 0.01.

**Table 4:** Pathways involved in genes whose expression correlates with time to parasitaemia.

Rank	Pathway name	Impact factor	#Genes in pathway	#Input genes in pathway	%Input genes in pathway	%Pathway genes in input	*P*-value
1	Leukocyte transendothelial migration	7.273	116	6	2.597	5.172	6.94E − 04
2	Calcium signaling pathway	5.203	175	6	2.597	3.429	0.0055
3	Natural killer cell mediated cytotoxicity	4.948	131	5	2.165	3.817	0.007099
4	Focal adhesion	4.694	195	6	2.597	3.077	0.009154
5	Jak-STAT signaling pathway	4.32	153	5	2.165	3.268	0.0133
6	Fc epsilon RI signaling pathway	3.465	75	3	1.299	4	0.031274
7	Phosphatidylinositol signaling system	3.398	77	3	1.299	3.896	0.033438
8	Regulation of actin cytoskeleton	3.162	208	5	2.165	2.404	0.042341
9	T cell receptor signaling pathway	2.929	93	3	1.299	3.226	0.053438
10	GnRH signaling pathway	2.828	97	3	1.299	3.093	0.05916

Genes identified as showing expression correlating with number of days to parasitaemia in experimental sporozoite challenge by Spearman's rank testing were analysed by Pathway Express for linkage in the literature to biological pathways.

**Table 5:** Modular analysis of genes positively and negatively enriched.

Study	Module	Enrichment—% of genes in module	Example genes	Fold change
*CSP study* (CSP stimulated versus unstimulated PBMC)	*1. Interferon induced*	30% positively enriched	*CCL8 CXCL10 WARS*	2.6× up 2.4× up 2.3× up
*2. Adipocytes*	29% positively enriched	*CD53 HCLS1 IFI30*	1.3× up 1.3× up 1.2× up
*TRAP* (TRAP stimulated versus unstimulated PBMC)	*1. Proteasome*	72% positively enriched	*PSME1 PSMC3 ADRM1*	1.4× up 1.3× up 1.2× up
*2. Interferon induced*	30% positively enriched	*CCL8 WARS STAT1*	5.6× up 3.0× up 2.7× up
*Protection* (3 subjects with complete protection versus 11 subjects with malaria)	*1. Interferon induced*	32% positively enriched	*CXCL10* *IFI35 TNFSF10*	2.9× up 1.7× up 1.6× up
*2. Antigen presentation*	14% positively enriched	*PLEK* *HLA-DQA1 ICAM1*	1.5× up 1.4× up × up
*1. Haemopoetic stem cell*	32% negatively enriched	*GNA15 DPYSL3 PDGF*	1.6× down 1.4× down 1.3× down
*2. Regulatory monocytes*	16% negatively enriched	*CXCL5 ABCG1 TRIP10*	2.6× down 1.9× down 1.8× down
*3. Myeloid lineage*	32% negatively enriched	*STAB1 TGFB1 CD59*	1.7× down 1.5× down 1.4× down

Genes were ranked according to their differential expression across two conditions and the gene lists generated underwent Gene Set Enrichment Analysis (GSEA) to quantify the degree to which the database genesets occur towards the top (up-regulated genes) or towards the bottom (down-regulated genes) of the ranked list of genes from the experiment. An enrichment score was assigned for each geneset using a variation of the Kolmogorov–Smirnov test and the enrichment scores were normalised for the size of the gene sets and correction for multiple testing by computing the Benjamini and Hochberg false discovery rate. Pathways up or down regulated were displayed by modular mapping, whereby each gene set was assigned to a module using online pathway databases and PubMed literature.
